# 1452. *Neuroinvasive Bacillus Cereus* Infection Amongst Immunocompromised Patients: Epidemiological Investigation of 5 Cases in Patients with Acute Myeloid Leukemia

**DOI:** 10.1093/ofid/ofad500.1289

**Published:** 2023-11-27

**Authors:** Jessica S Little, Cassie Coughlin, Candace C R Hsieh, Meaghan Lanza, Wanyi Huang, Aishwarya Kumar, Tanvi Dandawate, Robert Tucker, Marisa Winkler, Nicole Pecora, Marlise Luskin, Ann E Woolley, Nicolas C Issa, Lindsey R Baden, Christina Brandeburg, Barabara Bolstorff, Esther D Fortes, Matthew Doucette, Eileen McHale, Craig Bunnell, Dana Platt, Sonali Desai, Anne Gross, Karen Fiumara, Chanu Rhee, Michael Klompas, Meghan Baker

**Affiliations:** Brigham and Women's Hospital, Boston, Massachusetts; Dana-Farber Cancer Institute, Boston, Massachusetts; Dana-Farber Cancer Institute, Boston, Massachusetts; Dana-Farber Cancer Institute, Boston, Massachusetts; Dana Farber Cancer Institute, Newton, Massachusetts; Dana-Farber Cancer Institute, Boston, Massachusetts; Dana-Farber Cancer Institute, Boston, Massachusetts; Brigham and Women's Hospital, Boston, Massachusetts; Massachusetts General Hospital, Boston, Massachusetts; Brigham & Women's Hospital, Boston, Massachusetts; Dana-Farber Cancer Institute, Boston, Massachusetts; Brigham and Women's Hospital, Boston, Massachusetts; Brigham & Women's Hospital, Boston, Massachusetts; Brigham and Women's Hospital, Boston, Massachusetts; Massachusetts Department of Public Health, Boston, Massachusetts; Massachusetts Department of Public Health, Boston, Massachusetts; MA SPHL, Jamaica Plain, Massachusetts; Massachusetts Department of Public Health, Boston, Massachusetts; Massachusetts Department of Public Health, Boston, Massachusetts; Dana-Farber Cancer Institute, Boston, Massachusetts; Dana-Farber Cancer Institute, Boston, Massachusetts; Brigham and Women's Hospital, Boston, Massachusetts; Dana-Farber Cancer Institute, Boston, Massachusetts; Brigham and Women's Hospital, Boston, Massachusetts; Brigham and Women's Hospital, Boston, Massachusetts; Harvard Medical School and Harvard Pilgrim Health Care Institute, Boston, Massachusetts; Brigham and Women's Hospital, Boston, Massachusetts

## Abstract

**Background:**

*Bacillus cereus* can cause serious nosocomial infections, including neuroinvasive infections in immunocompromised patients. Three patients with AML developed hospital-onset neuroinvasive *B. cereus* infections in close temporal proximity at our institution in 2022, triggering an extensive epidemiological investigation.

**Methods:**

We identified all patients with AML, positive microbiologic assays for *Bacillus* species >48 hours after admission, and neurological symptoms with radiologic findings admitted between January 2018 and October 2022 (Figure 1). Infection control practices were observed, environmental samples obtained, and a dietary case-control study was performed. *Bacillus* isolates were sequenced, including 2 case isolates, 61 environmental specimens, and 19 samples of a protein supplement common to most AML patients.

**Figure 1.**
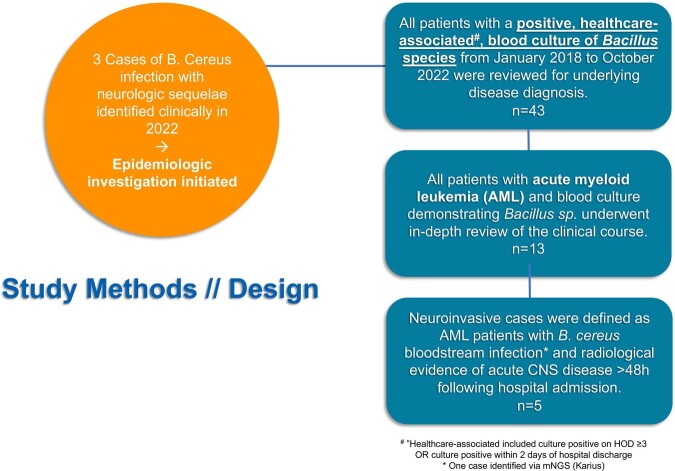
Case Identification and Definitions

**Results:**

Five AML patients with *B. cereus* neuroinvasive disease were identified. Four were identified via blood culture and one via metagenomic sequencing of plasma cell-free DNA. All patients were hospitalized for induction chemotherapy and were neutropenic (Table 1; Figure 2). Central nervous system findings included intraparenchymal and subarachnoid hemorrhage, and rim-enhancing lesions. All patients were treated with ciprofloxacin and survived without neurologic sequelae. *B. cereus* was positively identified in 7/61 environmental and 1/19 protein samples. There was no single exposure common to all patients per the dietary case-control study, hospital construction records, or environmental samples. Sequencing confirmed all isolates were unrelated. Ciprofloxacin was added to the empiric antimicrobial regimen for AML patients with neutropenic prolonged or recurrent fevers in September 2022; no new cases have since been identified through April 2023 (Figure 3).

**Table 1.**
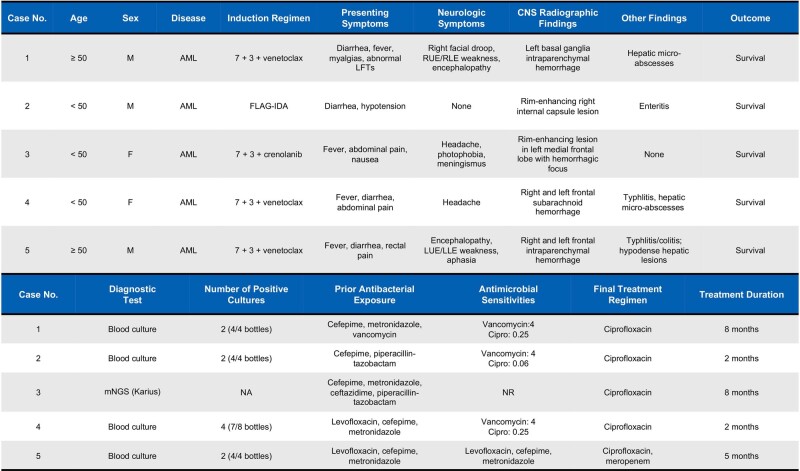
Clinical Characteristics of Neuroinvasive Bacillus Cereus Cases

**Figure 2.**
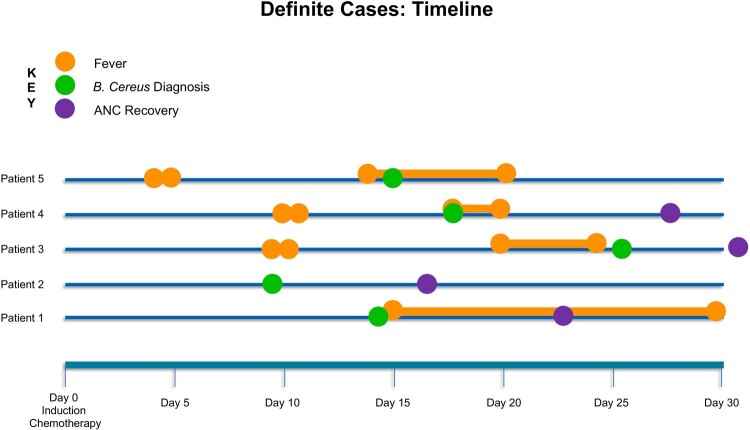
Timeline of Clinical Presentation of Neuroinvasive Bacillus Cereus Infection

**Figure 3.**
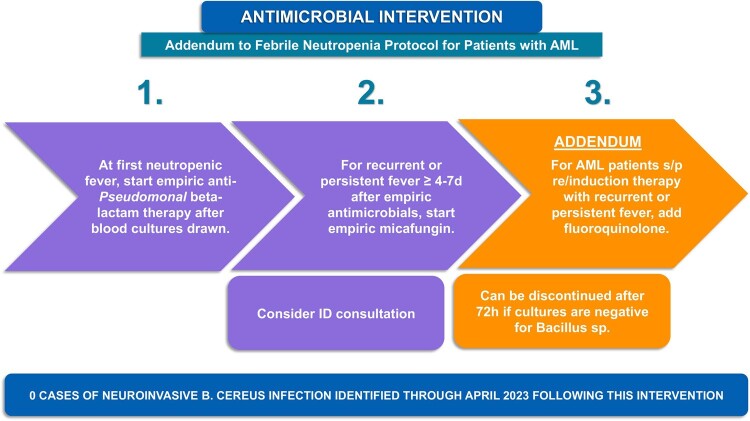
Antimicrobial Protocol for Prevention of Neuroinvasive Bacillus Cereus Infection

**Conclusion:**

*B. cereus* is ubiquitous in the hospital environment, sometimes leading to pseudoclusters with unrelated isolates. Fastidious infection control practices addressing a broad range of potential exposures are warranted to prevent nosocomial infections. Including *B. cereus* coverage in empiric regimens for AML patients with prolonged or recurrent neutropenic fever may prevent serious infections from this pathogen.

**Disclosures:**

**Nicolas C. Issa, MD**, AiCuris: Grant/Research Support|Astellas: Grant/Research Support|Boehringer Ingelheim: Advisor/Consultant|Fujifilm: Grant/Research Support|GSK: Grant/Research Support|Merck: Grant/Research Support|Moderna: Grant/Research Support **Chanu Rhee, MD, MPH**, Cytovale: Advisor/Consultant|Pfizer: Advisor/Consultant|UpToDate, Inc.: Honoraria **Michael Klompas, MD, MPH**, UpToDate, Inc.: Royalties for chapters on pneumonia

